# Intraocular toxicity caused by MEROCTANE perfluorocarbon liquid

**DOI:** 10.1038/s41598-020-79561-y

**Published:** 2021-01-12

**Authors:** Rosa M. Coco-Martin, Cristina Andrés-Iglesias, Girish K. Srivastava, Javier Lagos-Rodriguez, Miguel Ruiz-Tevah, Mario R. Díaz-Cárdenas, Ivan Fernandez-Bueno, Juan García-Serna, María T. García-Gutierrez, Alfredo García-Layana, J. Carlos Pastor

**Affiliations:** 1 Instituto Universitario de Oftalmobiología Aplicada (IOBA), Eye Institute, University of Valladolid, Paseo de Belen, 17, 47011 Valladolid, Spain; 2 Oftared, Carlos III National Institute of Health, Madrid, Spain; 3 Hospital Regional Liberador Bernardo O’Higgins and Clínica Vision, Rancagua, Chile; 4 Hospital San Borja Arriarán and Instituto de La Visión, Santiago, Chile; 5 Hospital Clínico and University of Magallanes, Punta Arenas, Chile; 6 High Pressure Process Group, Departamento de Ingeniería Química y Tecnología del Medio Ambiente, Instituto de Bioeconomía, University of Valladolid, Valladolid, Spain; 7 Department of Ophthalmology, Clínica Universitaria de Navarra and University of Navarra, Pamplona, Spain; 8 Department of Ophthalmology, Hospital Clínico Universitario, Valladolid, Spain

**Keywords:** Translational research, Mass spectrometry, Cell culture

## Abstract

Serious intraocular toxicity cases have been reported worldwide after the use of different perfluorocarbon liquids. The current study reports for the first-time the clinical pictures of cases of acute intraocular toxicity caused by MEROCTANE, a perfluoro-octane commercialized by a Turkish company and distributed in many countries. A series of 18 cases from Chile and Spain was retrospectively analysed. To evaluate the impurity profile, a suspicious MEROCTANE sample (lot OCT.01.2013) was analysed by gas chromatography mass spectrometry and compared with a non-suspicious sample of the same commercial perfluoro-octane (lot OCT 722011). Cytotoxicity was tested following a direct-contact method, taking into consideration the high volatility and hydrophobicity of perfluoro-octane and following the ISO 10993 guideline. Cytotoxicity test showed clear cytotoxic effects of the analysed batch (less than 9% of cell viability). Moreover, chemical analysis demonstrated the presence of many contaminants, some highly toxic (acids and alcohols). Perfluorocarbon liquids are useful tools for intraocular surgery but companies and Agencies of Medical Devices must implement measures that guarantee the safety of these products based on both chemical and cytotoxicity analysis for every batch**.** Medical staff should be encouraged to report any suspected case to their respective National Agencies.

## Introduction

In recent years serious incidents with the use of perfluorocarbon liquids (PFCL) used during intraocular surgery, mainly perfluoro-octane (PFO), have been reported, which has led to hundreds of blind eyes worldwide^1^. Several impurities have been identified in the toxic samples, and they have been linked to the high levels of toxicity^2–5^. Consequently, the use of highly purified PFOs with appropriate cytotoxic control remains the only safe option when using these compounds, which facilitate intra-surgical manoeuvres in many cases. These medical devices were “CE” marked before being sold in European Countries, assuming that they are correctly manufactured and appropriately tested according to ISO standards and European Medical Devices directives. These situations show that one or several of the currently accepted safety mechanisms failed, and the consequence was that many cases of toxicity appeared.

In 2015 our research group had the opportunity to deal with the problem caused by ALA OCTA (AlaMedics, Dornstadt, Germany), which was suspected of causing acute toxicity, and we were able to identify some of the toxic compounds^3^. Shortly afterwards, another incident occurred, this time with BIO OCTANE PLUS (Biotech Ophthalmology PVT Ltd, Gujarat, India), and we were also able to identify the toxic contaminants causing the problem^2,5^.

But the series of acute severe incidents began in 2013, with the report to the Spanish Agency of Medicines and Medical Devices (AEMPS) of four cases of blindness deriving from the use of MEROCTANE (Meran Tip Ltd, Istanbul, Turkey). In December 2013, the AEMPS removed the product from the Spanish market. Toxicity was evaluated by a direct contact method developed according to ISO 10993-5-2009 standard, demonstrating that one batch (OCT 07.2013) was clearly toxic. Chemical analysis made by a National Institute was unable to identify the toxic contaminants and only perfluorodecalin residues were found in the PFO, a finding which was interpreted as a lack of care by the manufacturer in the handling of both products.

Nevertheless, at the beginning of May 2013, cases of acute toxicity were reported in Chile after the use of this commercial product, and the Chilean Medicines Agency launched a “sanitary” alarm, on August 30th, 2013, recommending the use of this PFO be terminated and its commercialization be prevented in that country.

Recently, we have been able to access a sample of one of the toxic batches of MEROCTANE and to analyse it, finding a chromatographic profile with a significant number of impurities where some compounds have previously shown toxicity in other toxic PFOs. Also, we have been able to analyse the clinical information coming from 18 cases.

Since we have not found published references to MEROCTANE toxicity and its clinical consequences, the purpose of this paper is to report these original findings. We consider that the information, on this very serious adverse event, is of the utmost relevance, affecting several countries, and we must do everything possible to prevent such incidents from recurring.

We also want to emphasize the need to combine appropriate chemical analysis with effective cytotoxicity tests, and always by batches. Similarly, we stress the need for ophthalmologists to report to their respective Medical Devices Agencies suspicion of any adverse reaction with any product. Finally, the Adverse Effects Alert Systems for Medical Devices of different countries worldwide (not only those of the European Union) must be improved, with faster and more efficient coordination.

## Results

### Clinical features

#### Spanish cases

**Figure 1:**
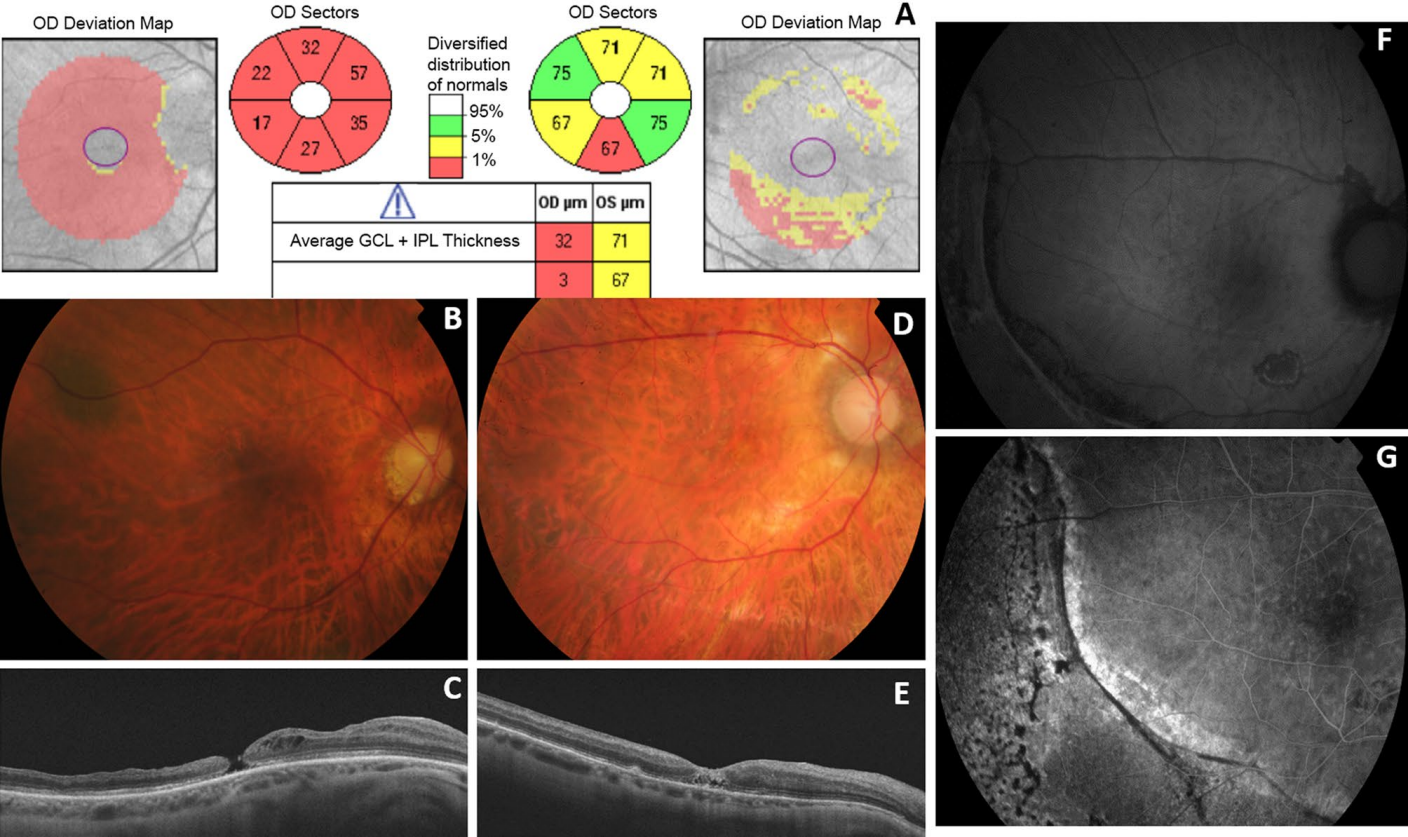
Clinical pictures from two Spanish patients. One Spanish patient showed evidence of optic nerve atrophy on the OCT (**A**) and in the ocular fundus (**B**), and a postoperative macular hole (**C**). On the right-hand side, the fundus of another Spanish patient showed signs of retinal and optic nerve atrophy and highly stenotic vessels (**D**); there is foveal disruption on the OCT (**E**) and pigmentary changes at the edge where octane contacted with the retina is also evident on autofluorescence (**F**) and angiofluorescein images (**G**).

Four consecutive patients underwent uneventful vitreoretinal surgery by one experienced surgeon (AGL) in a sole Spanish center, using MEROCTANE (batch OCT.07.2013). Three were operated on for a retinal detachment and one due to a cataract complication. In the immediate post-operative period, each patient presented features compatible with severe acute retinal toxicity^3^. None presented postoperative IOP increase. There was no inflammation, vasculitis, or bleeding in any of them. A few weeks after surgery retinal thinning, highly narrow vessels and atrophy of the optic nerve were observed (Fig. 1). Subretinal pigmentary changes at the edge where the intraoperative PFO bubble contacted with the retina were also evident by autofluorescence and angiofluorescein images. They all had foveal disruption and one patient developed a macular hole that had an extremely thick inner limiting membrane (ILM) when he was re-operated on (Fig. 1). Final postoperative Visual Acuity (VA) was very poor in all cases. Decreased amplitude of “a” and “b” waves were observed in the photopic and scotopic electroretinogram in the operated eye.

#### Chilean cases

Fourteen consecutive patients underwent uneventful vitreoretinal surgery by experienced surgeons (JLR, MRT, MRDC) from 3 different clinical centers (Rancagua, San Borja and Punta Arenas), using MEROCTANE, batch number OCT.01.2013. In the immediate postoperative period each patient showed clinical features compatible with severe acute retinal toxicity^3^.

**Figure 2:**
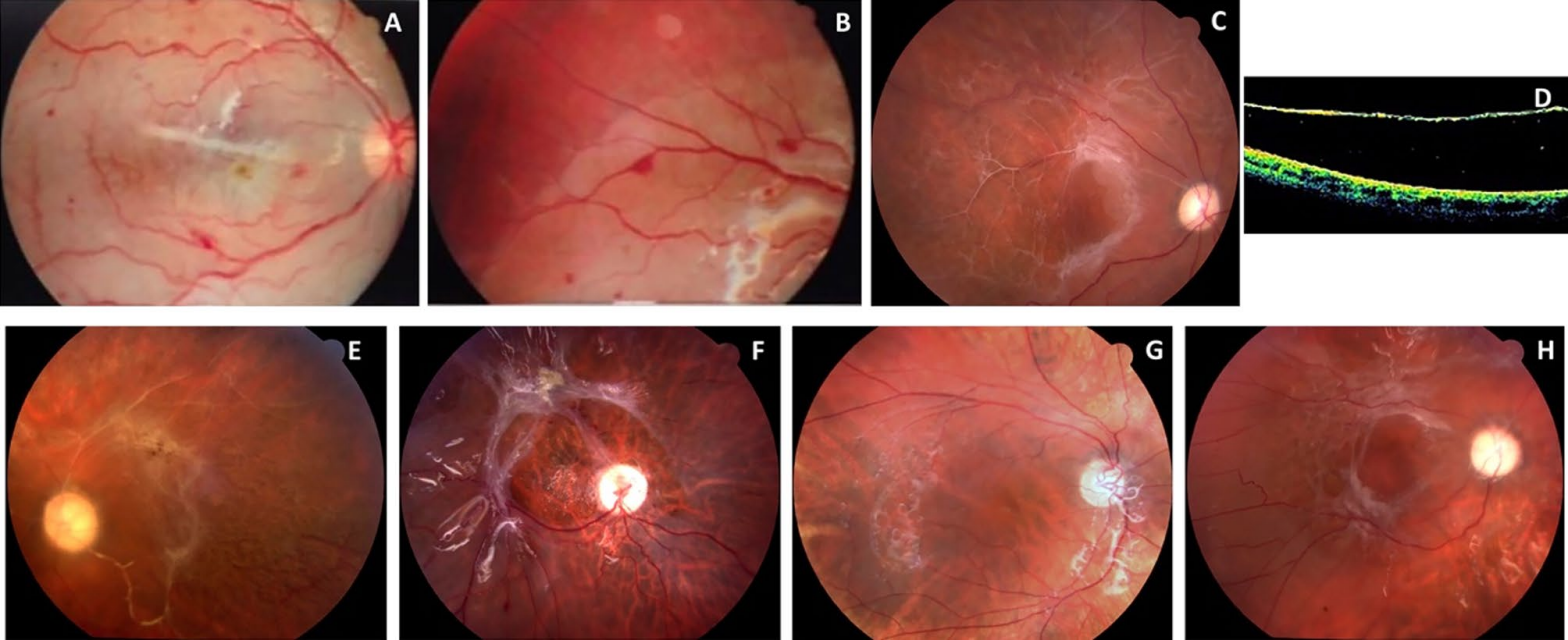
Clinical pictures from six Chilean patients. Retinographies from the Punta Arenas’ case showed evidence of retinal necrosis and small intraretinal haemorrhages at the posterior pole (**A**) and the edge was at the border of the PFO filling (**B**). The other images are from San Borja cases. They all display optic nerve atrophy. Case 1 shows PVR with epiretinal membranes (**C**) and thin retina detached on OCT (**D**). Case 2 looks similar to Case 1, with even more ghost vessels (**E**). Case 3 shows proliferative vitreoretinopathy with thick epiretinal membranes and a huge hole at the posterior pole (**F**). Case 4 displays signs of retinal atrophy (**G**). Case 5 shows epiretinal membranes and a huge macular hole (**H**).

No patient presented postoperative IOP increase during the following days. Clinical data are summarized in Table 1 and Fig. 2.

**Table 1:** Clinical data.

Case	Sex/age	Pathology (eye)	VA preop–postop	Early postop	Late postop
Spain 1	M / 48 yrs	RD (LE)	20/125–< 20/800	ON atrophy, retinal thinning and retinal pigment epithelium atrophy. RD relapse needing VPP and SiO injection	Reattached retina
Spain 2	W /57 yrs	RD (RE)	20/32–20/250	Retinal thinning, macular hole and ERM. RD relapse needing VPP and scleral buckling	Reattached retina
Spain 3	M / 72 yrs	RD (RE)	20/32–20/800	Retinal thinning, ON and retinal pigment epithelium atrophy	Reattached retina
Spain 4	M / 90 yrs	Posterior capsule rupture due to cataract surgery (RE)	20/20–HM	Retinal thinning, ON and retinal pigment epithelium atrophy	Reattached retina
Chile-Rancagua 1	W /65 yrs	RD (LE)	20/40–NPL	PVR, retinal ischemia and necrosis. RD relapsed needing buckle and SiO injection. Retina very friable during reintervention	Phthisis bulbi
Chile-Rancagua 2	M / 70 yrs	RD (LE)	HM–NPL	ON atrophy and ischemia. Maximum IOP 32 mmHg	Reattached retina
Chile-Rancagua 3	M /62 yrs	RD (LE)	PL–NPL	Massive PVR, retinal ischemia and necrosis. RD relapsed needing SiO injection	Reattached retina
Chile-Rancagua 4	M / 59 yrs	RD (RE)	20/60–NPL	PVR and RD relapse. Maximum IOP 37 mmHg	Phthisis bulbi
Chile-Rancagua 5	M /62 yrs	RD due to PDR + retinal tear (RE)	CD–NPL	ON atrophy and retinal ischemia of the posterior pole	Phthisis bulbi
Chile-Rancagua 6	M / 42 yrs	RD (RE)	PL–NPL	Corneal edema. Maximum IOP 24 mmHg. ON atrophy and retinal ischemia with ghost vessels	Reattached retina
Chile-Rancagua 7	M / 68 yrs	RD (LE)	PL–NPL	ON atrophy and retinal ischemia with ghost vessels. RD relapsed needing SiO injection	Reattached retina
Chile-San Borja 1	M / 65 yrs	RD (RE)	HM–PL	Retinal thinning secondary to ischemia, ON atrophy and ERM. RD relapsed needing SiO injection	Reattached retina
Chile-San Borja 2	M / 54 yrs	RD and PVR (LE)	HM–NPL	Retinal Ischemia. Fibrine in the anterior chamber Maximum IOP 32 mmHg. ON atrophy and ERM	Reattached retina Pre phthisis
Chile-San Borja 3	W /63 yrs	RD (RE)	20/80–NPL	PVR with a big macular hole and thick ERMs. RD relapsed needing SiO injection	Rubeosis and RD relapsing Hyphemas. Phthisis bulbi
Chile-San Borja 4	M / 59 yrs	RD (RE)	CF–NPL	Retinal thinning and ON atrophy. Maximum IOP 28 mmHg	Reattached retina
Chile-San Borja 5	W /36 yrs	RD (RE)	CF–PL	PVR with a big macular hole and thick ERMs. ON atrophy. Maximum IOP 16 mmHg. RD relapsed needing SiO injection Cataract that needed reintervention associated to PPV	Reattached retina
Chile-San Borja 6	W /60 yrs	RD relapse (RE)	CF–NPL	ON atrophy. Maximum IOP 11 mmHg. RD relapsed needing SiO injection. Patient needed 3 PPV	Reattached retina
Chile-Punta Arenas 1	M / 30 yrs	Infectious panuveitis and RD (LE). VIH ( +)	PL–NPL	White retina with a clear edge where the PFO contacted. PVR & RD relapse	Irreparable RD

Batch numbers from Chile: OCT.01.2013 for all cases. Batch from Spain was OCT.07.2013. The last two cases were initially operated with PPV + SiO, in all others PPV + gas was performed as primary surgery.

*VA* visual acuity, *preop* preoperative, *postop* postoperative, *RD* retinal detachment, *PDR* proliferative diabetic retinopathy, *PVR* proliferative vitreoretinopathy, *M* male, *W* woman, *RE* right eye, *LE* left eye, *CF* counting fingers, *HM* hand movements, *CF* counting fingers, *PL* perception of light, *NPL* no perception of light, *SiO* silicon oil, *ON* optic nerve, *IOP* intraocular pressure, *ERM* epiretinal membrane, *PPV* pars plana vitrectomy.

### Cytotoxicity evaluation

All quality standards from ISO 10993-5 (biological evaluation of medical devices) were met for the cytotoxicity tests. Cell cultures for biological analysis should respond to the positive control sample (phenol; 0–1% viability), and cell cultures incubated with culture medium control samples (100% viability) with a number of homogeneous cells maintained ≤ 15% variation in each culture plate and, finally, OD > 0.2. The cell cultures exposed to one non-toxic PFO negative control sample maintained viability between 95 and 99% compared to the 100% viable cell cultures incubated with the cell culture medium.

**Figure 3:**
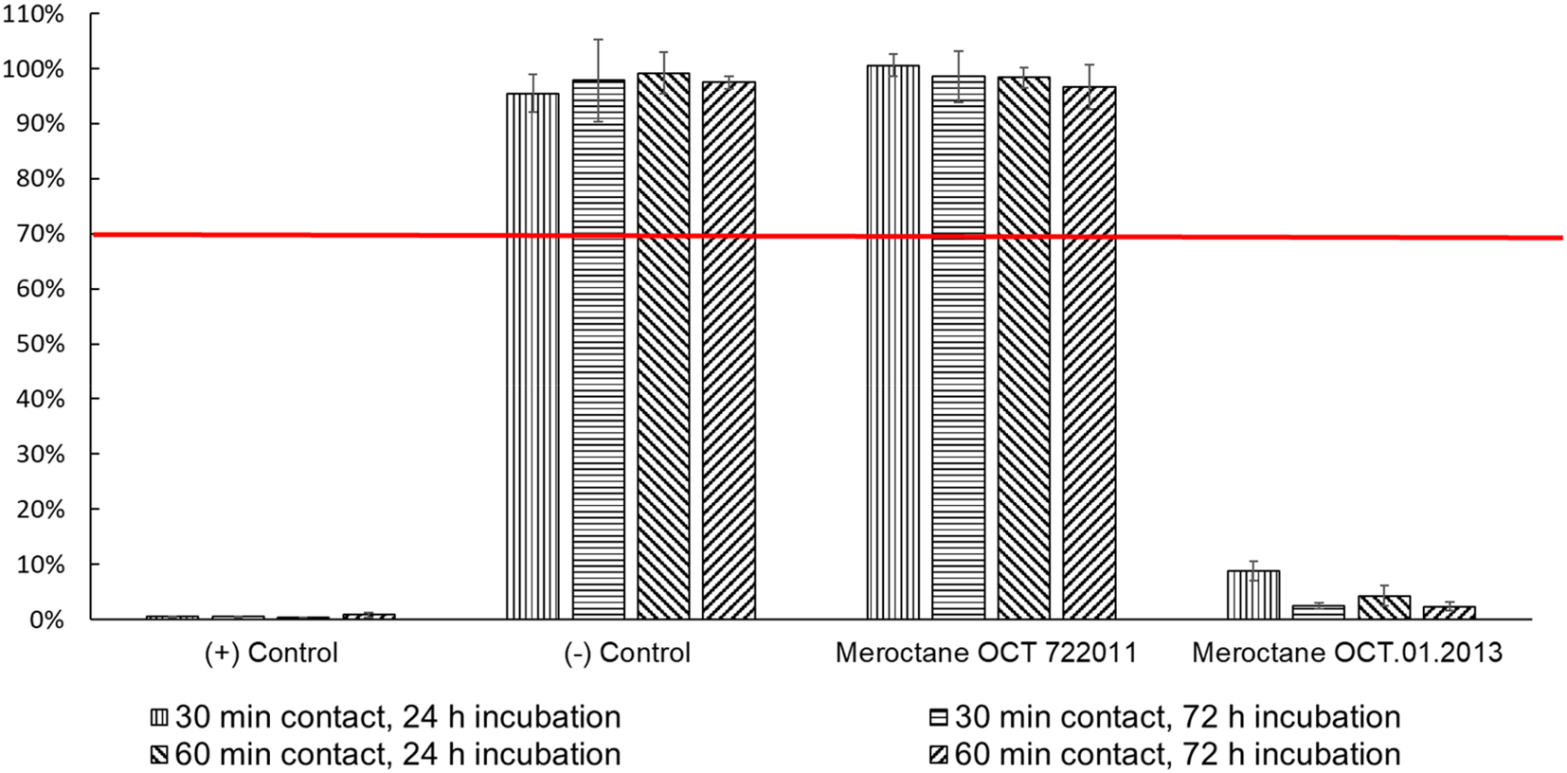
Cytotoxicity evaluation of MEROCTANE batches OCT 722011 and OCT.01.2013. Samples with ≤ 70% viability were considered cytotoxic (ISO 10993-5:2009). PFO batch OCT 722011 was distributed and used in Spain, no acute toxic cases were reported. PFO batch OCT.01.2013 was distributed in Chile causing acute toxicity in patients.

Cell viability was evaluated after 30 and 60 min exposure time, and 24 and 72 h incubation time after exposure. As it is shows in Fig. 3, in all the experiments, MEROCTANE batch OCT.01.2013 was extremely cytotoxic for cell cultures, considering that values below 70% viability are deemed cytotoxic according to ISO-10993-5. Cell viability was reduced to 9% ± 1.8% (30 min exposure, 24 h incubation), 3% ± 0.6% (30 min exposure, 72 h incubation), 4% ± 1.8% (60 min exposure, 24 h incubation) and 2% ± 0.8% (60 min exposure, 72 h incubation). However, MEROCTANE batch OCT 722011 did not display cytotoxicity, showing cell viability values of 100% ± 2.0% (30 min exposure, 24 h incubation), 99% ± 4.6% (30 minues exposure, 72 h incubation), 98% ± 1.8% (60 min exposure, 24 h incubation), and 97% ± 4.1% (60 min exposure, 72 h incubation).

### Chemical analysis

**Figure 4:**
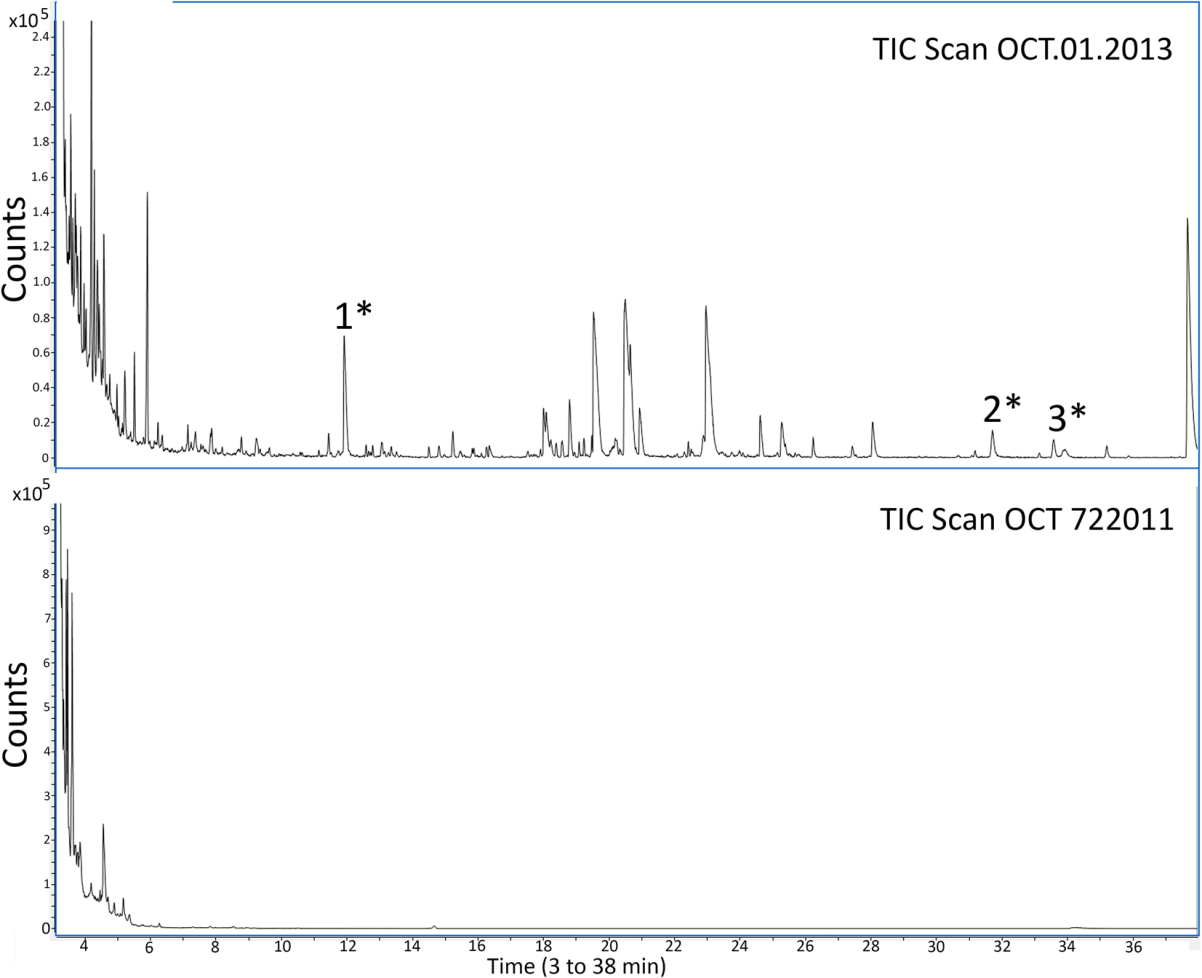
MEROCTANE samples total ion chromatograms (TIC) of from 3 to 38 min (Agilent Mass Hunter qualitative analysis B.07.00). PFO batch OCT 722011 was distributed and used in Spain, no acute toxic cases were reported. PFO batch OCT.01.2013 was distributed in Chile causing acute toxicity in patients. (1) 1,1,1,2,2,3,3,4,4,5,5,6,6-Tridecafluorotridecane, C_13_H_15_F_13_ (2) 1H,1H,7H-Dodecafluoro-1-heptanol, C_7_H_4_F_12_O (3) 1H,1H,9H-Hexadecafluoro-1-nonanol, C_9_H_4_F_16_O. (*) Identification with Match and R. Match above 700.

**Figure 5:**
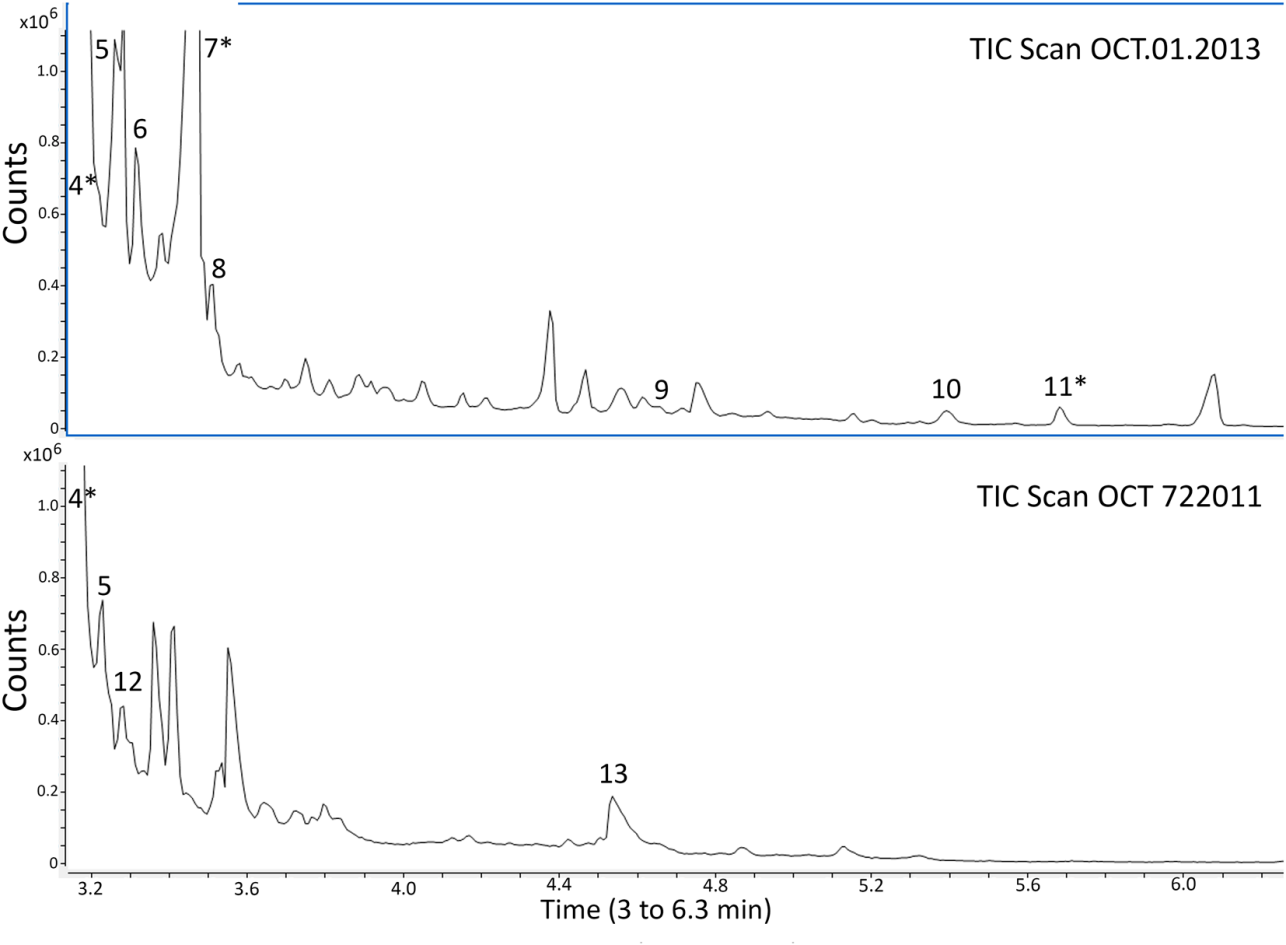
MEROCTANE samples total ion chromatograms (TIC) amplification from 3 to 6.3 min (Agilent Mass Hunter qualitative analysis B.07.00). PFO batch OCT 722011 was distributed and used in Spain, no acute toxic cases were reported. PFO batch OCT.01.2013 was distributed in Chile causing acute toxicity in patients. (4) Perfluorodecalin, C_10_F_18_ (5) 1,1,1,2,2,3,3,4,4,5,6,6,6-Tridecafluorohexane, C_6_HF_13_(6) 1H,1H,11H-Eicosafluoro-1-undecanol, C_11_H_4_F_20_O (7) Perfluoro-N-(4-methylcyclohexyl) piperidine, C_12_F_23_N (8) Perfluorododecanoic acid, C_12_HF_23_O_2_ (9) Pentadecafluoro octanoic acid, methyl ester, C_9_H_3_F_15_O_2_ (10) 1,1,1,3,3,3-Hexafluoropropane, C_3_H_2_F_6_ (11) Pentadecafluorooctanoic acid, isopropyl ester, C_11_H_7_F_15_O_2_ (12) Methyl 2,2,3,3,4,4,5,5,6,6,7,7-dodecafluoroheptanoate, C_8_H_4_F_12_O_2_ (13) 1H,1H-Perfluoro-1-heptanol, C_7_H_3_F_13_O. (*) Identification with Match and R. Match above 700.

Analysis by GC–MS showed that both MEROCTANE batches OCT 722011 and OCT.01.2013 had many impurities when compared to the PFO control, where no impurities were found (data not shown). As can be seen in Figs. 4 and 5, when both MEROCTANE samples were compared, the OCT.01.2013 sample had many more impurities than MEROCTANE OCT 722011. From all the impurities it was possible to identify some of them as perfluorodecalin, one acid, several alcohols or one ester, among others.

## Discussion

This paper shows the severe clinical effects of the use of toxic PFO MEROCTANE, which are similar to other toxic events already reported with other PFCLs^3,5^. In addition, and although chemical findings must be interpreted with some caution since the analysed sample had exceeded its expiry date, at least one of the contaminants identified in the toxic batch of MEROCTANE (1H,1H,7H-Dodecafluoro-1-heptanol) has been previously described in ALA OCTA toxic samples^2^. However, in this case, we did not find benzene derivatives or any other leachable substances that might come from the vials.

Although clinical toxicity seems to be different from batch to batch, with the Spanish cases being less severe than those from Chile, most of the patients showed a clear retinal atrophy evidently related to the area of contact with PFO during surgery. Mention should be made of the high percentage of cases developing *phthisis bulbi* in the Chilean series, compared to previous reports of another toxic PFCL (ALA OCTA or BIO OCTANE PLUS)^3^. In all cases, the manufacturers gave the assurance that the purity of their product was greater than 99%, which reaffirms our idea that it is critical to determine the type of contaminants and not only the degree of purity. It seems that these particular contaminants produced serious effects even at very low concentrations.

An analysis of the safety flaws that have caused these catastrophes is very informative. Evidently, the company evaluated the safety of these medical devices by performing tests before their commercialization. These tests were carried out under ISO standards and European Regulations, which are necessary to obtain CE certification.

In fact, at the request of the Health Authorities, they showed certificates made by notified bodies; in the case of the European market, from the company "Electrotechnical Testing Institute" of the Czech Republic.

Yet one of the problems, already highlighted by our group in different publications^1–3^, is that ISO standards do not adequately define the test conditions. They should take into account the particular chemical characteristics of these products, such as high volatility and hydrophobicity of the PFCL when testing these compounds. These critical aspects have already been incorporated into the new version of ISO 16672:2020 “Ophthalmic implants—Ocular endotamponades (https://www.iso.org/standard/70806.html).

The other problem is related to the need to evaluate every lot, an aspect which many companies are reluctant to do, arguing that once a batch has been evaluated, the same result will apply to the others. However, the evidence that toxicity is strongly batch-dependent has been previously demonstrated in ALA OCTA^3^, BIO OCTANE PLUS^2^, and now with MEROCTANE.

Thus, in this paper, two batches of MEROCTANE have been analysed, showing very different profiles regarding impurities and toxicity. Also, we have obtained information on other batches analysed at different times by our group and by the Spanish Agency of Medicine and Medical Devices (AEMPS). In brief, and regarding this information, batches OCT.01.2013 (the one tested for this paper), OCT.07.2013 and OCT.53.2011 were highly toxic, while batches OCT 722011 and OCT.11.2012 (unpublished data) were not. It is possible that the differences in toxicity are due to differences in the production methods of the PFCL, information to which we have not had access, as it depends on manufacturers usually located outside the European Union that do not provide this information. But other reasons cannot be ruled out, since in the case of ALA OCTA a clear influence of the storage time before packaging on an increase in cytotoxicity was demonstrated^3^.

These, and other examples with other medical devices for intraocular use, have resulted in our insistence that, in addition to chemical analyses ensuring an acceptable degree of purity, companies should be forced to carry out cytotoxicity studies on each batch. Toxicity, in our opinion, is not a simple matter related to the percentage of purity (obviously, the purer the better) but mainly to the nature of the contaminants, which, depending on the compound, can be highly cytotoxic even at very low concentrations; this has been demonstrated for acids or side products from manufacturing as in the case of BIO OCTANE PLUS^2,6^. Table 2 summarizes information on reported toxicity cases of PFO from the three different manufacturers, Meran, AlaMedics and Biotech.

**Table 2:** Summary of PFO cases.

Product	Company	Origin	Outbreack years	Affected patients in Spain	Other countries affected^a^	Cytotoxic tested batches	% Cell mortality^d^	Identified impurities	References
MEROCTANE	Meran	Turkey	2013	4	Chile^c^	OCT.01.2013	91	Tridecafluorotridecane	^10^
								1H,1H,7H-Dodecafluoro-1-heptanol	
								1H,1H,9H-Hexadecafluoro-1-nonanol	
								Tridecafluorohexane	
								1H,1H,11H-Eicosafluoro-1-undecanol	
								Perfluorododecanoic acid	
								Pentadecafluoro octanoic acid, methyl ester	
								Pentadecafluorooctanoic acid, isopropyl ester	
ALA OCTA	Alamedics	Germany	2014–2015	117	France	171214	99	Leachables (dimethylbenzene, ethylbenzene, p-xylene)	^2–5,8–10^
					Italy	061014	99	Perfluorooctanoic acid	
					Switzerland^b^	070714	47	1H,1H,7H-Dodecafluoro-1-heptanol	
					Sweden	050514	50	Underfluorinated impurities	
					Saudi Arabia	200114	44	Perfluorinated alkenes	
						180214	41	Perfluororurane	
						150414	66	1H-Perfluoroalkane analog	
						080714	47	1H-Perfluorooctane	
								Perfluorooctanoic acid methyl ester	
BIO OCTANE PLUS	Biotech Opthalmology PVT	India	2016	4		1605148	99	Tri-n-butyltinbromide	^2,5,10^

^a^Cases recognized by clinicians in different meetings and in some publications, but without access to official data from the National Health Agencies.

^b^At least 48 cases recently published (see references).

^c^Although they could not be included in this publication due to lack of data, there is evidence of at least 24 cases in Chile.

^d^Determined by the direct method developed by the IOBA (see references).

The last aspect that we would like to emphasize is the collaboration of both clinicians and National Medicines Agencies. The difference in legislation in some countries means that not all ophthalmologists are predisposed to declare their suspicious cases to their respective National Agency, a fact that is essential to detect and control new problems. When we reported the first cases of patients in Spain (Euretina Meeting, Copenhagen, 8–11 September 2016), some companies began to publicize in congresses that it was a Spanish problem. And although some of our colleagues reported similar cases^7^, it has been necessary to identify cases in other countries to eliminate that idea of the nationality of the problem. This is why publications like a recent paper by a group from Switzerland, recognizing 48 cases of toxicity with ALA OCTA that they had between April 2014 and October 2015, are crucial for the sake of this field^8^. Here, we would like to highlight that timing is everything when publishing these kinds of events. It is paramount for the results to be released as soon as they happen, and for us to have information on them.

Moreover, on August 30, 2013, the Department of Health Action, Sub-Department of Pharmacy of the Government of Chile issued a national alert recommending Chilean ophthalmologists to report their suspicious cases to their National Drug Agency. In spite of this, few official notifications have been produced, and our information regarding MEROCTANE has been followed in only two countries. In fact, this current paper is the first to offer clinical information together with biological and chemical analysis. According to information from the company, in 2013, 590 samples had been distributed in Chile and more than 16,000 worldwide (unconfirmed personal information). It is possible, therefore, that there are many more cases than those declared, as occurred with ALA OCTA.

Regarding the collaboration of Health Agencies, we urge that the information exchange should be improved worldwide. It does not seem appropriate that a product declared toxic in one country (August 2013) could be used almost four months later in another country, especially when the former issued an official "health alert". Toxic events in Spain occurred in September and October 2013.

In summary, we have decided to carry out this work for several reasons.

First, to provide fresh information on the impurities profile of a PFO from a Turkish company and foreseeably causing toxicity, a finding hitherto unknown. Second, to describe the clinical pictures of the affected patients, never previously reported. And third, to emphasize the importance of performing the appropriate cytotoxicity tests regarding the nature of the substance to be tested, and not only for complying with ISO standards.

Finally, to highlight the need for improved communication between National Medical Devices Agencies across countries worldwide and, in addition, for these agencies to encourage ophthalmologists (or any other health professional) to be willing to report suspected cases of any type of toxicity without having to fear an immediate charge of guilt.

## Methods

### Patients’ clinical examinations

A retrospective, descriptive case series study of MEROCTANE retinal toxicity is presented, including cases from Chile and Spain. This study followed the tenets of the Helsinki Declaration of 1964 (last amendment, 2013). The Clinical Research Ethics Committee of the Valladolid East Health Area approved the study with appropriate participants’ informed consent.

All clinical information was collected retrospectively looking for acute unexpected events, Data on both functional and structural status of the retina, was evaluated by RCM and JCP according to previously described clinical signs of PFO toxicity after the ALA OCTA episode^3^**.** The numbers of the MEROCTANE batches correspond to the date of packaging and the ones involved in this episode ranged from OCT.01.2013 to OCT.07.2013.

### Chemical and biological analysis

#### Samples

One non-toxic PFO sample currently marketed and repeatedly tested as non-toxic by our group was used as control (Control A). In addition, two samples of original packaged MEROCTANE from two different batches were analysed: batch number OCT 722011 (apparently nontoxic) and batch number OCT.01.2013, used on the patients with acute toxicity in Chile.

#### Gas chromatography–mass spectrometry analysis (GC–MS)

Control A and both MEROCTANE samples were analysed by GC–MS in triplicate. One µL of each sample was injected into a 7820A GC system gas chromatography (Agilent Technologies, USA), coupled to a 5977E MSD (Agilent Technologies, USA) single quadrupole mass spectrometer. For gas chromatographic separation a DB-Wax capillary column of 30 m × 0.25 mm × 0.25 μm (Agilent Technologies, USA) was used.

Detection and data acquisition were performed in scan mode from 20 to 700 Da. Data analysis was performed using Mass Hunter Data Acquisition software (Quantitative Analysis B.07.00, Agilent Technologies, USA) and tentative identification of the impurities was carried out using the NIST17 MS search 2.3 library and the EIC of the characteristic ions. The NIST MS Search parameter R. Match of the identified compounds was above 600 in all cases.

#### Cytotoxicity analysis

Direct contact cytotoxicity tests were performed as previously described by our group^5,9^. In brief, cultures of human retinal pigment epithelial cell line-19 (ARPE-19) cells were prepared in 96-well culture plates, followed by 24 h cell cycle synchronization in an FBS-free cell culture medium. The cultures were then exposed directly to the samples for 30 and 60 min. After exposure, the samples and the culture medium were removed from each well. The cell cultures were washed twice to remove any remnants and then incubated for 24 h and 72 h for cell growth. Subsequently, the viability of the cell cultures was measured by MTT assay^9^.

All experiments were performed in accordance with ISO guidelines for cell cytotoxicity tests and under Good Laboratory Practices certification. Viability values < 70% were considered cytotoxic (ISO 10993-5). Data were analysed by calculating the optical density (OD) value of cell culture viability in each well, which was recorded with a SpectraMax M5. The percentages of viable cells and standard deviations (SD) were calculated in Microsoft Excel^2,9^.
